# Characterization and Comparison of Bacterial Communities of an Invasive and Two Native Caribbean Seagrass Species Sheds Light on the Possible Influence of the Microbiome on Invasive Mechanisms

**DOI:** 10.3389/fmicb.2021.653998

**Published:** 2021-08-03

**Authors:** Tania Aires, Tamara M. Stuij, Gerard Muyzer, Ester A. Serrão, Aschwin H. Engelen

**Affiliations:** ^1^Centro de Ciências do Mar (CCMAR), Centro de Investigação Marinha e Ambiental (CIMAR), Universidade do Algarve, Faro, Portugal; ^2^CESAM - Centre for Environmental and Marine Studies, Department of Biology, University of Aveiro, Aveiro, Portugal; ^3^Microbial Systems Ecology, Department of Freshwater and Marine Ecology, Institute for Biodiversity and Ecosystem Dynamics, University of Amsterdam, Amsterdam, Netherlands; ^4^CARMABI Foundation, Willemstad, Curaçao

**Keywords:** seagrasses, invasions, adaptation, host–microbe interaction, drivers of microbiome, marine holobionts

## Abstract

Invasive plants, including marine macrophytes, are one of the most important threats to biodiversity by displacing native species and organisms depending on them. Invasion success is dependent on interactions among living organisms, but their study has been mostly limited to negative interactions while positive interactions are mostly underlooked. Recent studies suggested that microorganisms associated with eukaryotic hosts may play a determinant role in the invasion process. Along with the knowledge of their structure, taxonomic composition, and potential functional profile, understanding how bacterial communities are associated with the invasive species and the threatened natives (species-specific/environmentally shaped/tissue-specific) can give us a holistic insight into the invasion mechanisms. Here, we aimed to compare the bacterial communities associated with leaves and roots of two native Caribbean seagrasses (*Halodule wrightii* and *Thalassia testudinum*) with those of the successful invader *Halophila stipulacea*, in the Caribbean island Curaçao, using 16S rRNA gene amplicon sequencing and functional prediction. Invasive seagrass microbiomes were more diverse and included three times more species-specific core OTUs than the natives. Associated bacterial communities were seagrass-specific, with higher similarities between natives than between invasive and native seagrasses for both communities associated with leaves and roots, despite their strong tissue differentiation. However, with a higher number of OTUs in common, the core community (i.e., OTUs occurring in at least 80% of the samples) of the native *H. wrightii* was more similar to that of the invader *H. stipulacea* than *T. testudinum*, which could reflect more similar essential needs (e.g., nutritional, adaptive, and physiological) between native and invasive, in contrast to the two natives that might share more environment-related OTUs. Relative to native seagrass species, the invasive *H. stipulacea* was enriched in halotolerant bacterial genera with plant growth-promoting properties (like *Halomonas* sp. and *Lysinibacillus* sp.) and other potential beneficial effects for hosts (e.g., heavy metal detoxifiers and quorum sensing inhibitors). Predicted functional profiles also revealed some advantageous traits on the invasive species such as detoxification pathways, protection against pathogens, and stress tolerance. Despite the predictive nature of our findings concerning the functional potential of the bacteria, this investigation provides novel and important insights into native vs. invasive seagrasses microbiome. We demonstrated that the bacterial community associated with the invasive seagrass *H. stipulacea* is different from native seagrasses, including some potentially beneficial bacteria, suggesting the importance of considering the microbiome dynamics as a possible and important influencing factor in the colonization of non-indigenous species. We suggest further comparison of *H. stipulacea* microbiome from its native range with that from both the Mediterranean and Caribbean habitats where this species has a contrasting invasion success. Also, our new findings open doors to a more in-depth investigation combining meta-omics with bacterial manipulation experiments in order to confirm any functional advantage in the microbiome of this invasive seagrass.

## Introduction

In both terrestrial and aquatic environments, invasive plant and macrophyte species pose a direct threat to the biodiversity of their newly acquired habitat (Boudouresque and Verlaque, 2002; Sharma et al., 2005). In general, newly introduced species are regarded as invasive when, as a consequence of their influence (direct or indirect) on the natural habitat and food chain, the diversity and abundance of native species (plants or other organisms depending on them for food/shelter/nursery, etc.) are decreased. The fundamental causes of a switch to invasive behavior are still poorly understood (Sakai et al., 2001), but several factors are predicted to play a role such as life-history traits, genetic diversity of the imported pool, and interactions among living organisms. The “Enemy Release Hypothesis”(ERH) [e.g., (Keane and Crawley, 2002)], one of the most explored theories when it comes to invasions, assumes that, without their natural enemies (i.e., predators/parasites), the introduced species will invest in growth and reproduction, therefore displacing the native species. Most of the studies on invasive species explore the negative biotic interactions (e.g., predation and competition), whereas positive interactions are mostly underlooked, especially those promoted by microorganisms (often considered pathogens). Only recently have these been started to be explored by a few studies, suggesting that microorganisms can play a determinant role in invasions (Klock et al., 2015; Aires et al., 2016; Arnaud-Haond et al., 2017). Some of those microbe–host positive interaction and its possible role in the invasion process will be detailed below.

Invasion events and mechanisms of colonization of non-native terrestrial plants and marine macrophytes (i.e., seaweeds) are globally reported and widely studied (reviewed by Pyšek et al., 2012; Fleming and Dibble, 2015; Maggi et al., 2015). In contrast, to our knowledge, only three seagrass species, *Halophila ovalis*, *Zostera japonica*, and *Halophila stipulacea*, were found outside their native range, but only the last two have become successful after introduction beyond their native range (Lipkin, 1975; Harrisson and Bigley, 1982; Ruiz and Ballantine, 2004; Short et al., 2010). Yet, there is still an ongoing debate on whether these non-native seagrasses are truly invasive. *Z. japonica*, whose native range lies along the Western Pacific coast, i.e., from Vietnam to Northern Russia, was documented for the first time in Washington State in 1957 (Harrisson and Bigley, 1982) and might have been introduced by the transportation of Pacific oysters from Japan to the United States (Wonham and Carlton, 2005; White et al., 2009). Nowadays, it occurs widely along the west coast from British Columbia to Southern Oregon, where it colonized naturally unvegetated tidal flats. This increase in *Z. japonica* did not result in a decreased abundance of the native *Zostera marina*, suggesting that competition between the two seagrasses is low due to habitat differentiation (Gaeckle et al., 2011).

*Halophila stipulacea* is native in the Red Sea, Persian Gulf, and the Indian Ocean (den Hartog, 1970) and was spotted for the first time in the Mediterranean Sea in 1895 (Fritsch, 1895) being considered one of the first Lessepsian migrants. The extremely early record right after the opening of the Suez Canal and its fast and successful spread and establishment led some authors to believe that, instead, *H. stipulacea* was a paleo Mediterranean relic that survived the late Miocene crisis (Taviani, 2002). However, other authors (Por, 1971) defended that this species relied on its great adaptability to the salinity barriers across the Canal to spread along the Mediterranean Sea. This seagrass is nowadays found all over the Mediterranean Sea (Gambi et al., 2009; Sghaier and Zakhama-sraieb, 2011; Winters et al., 2020), but its effect on native seagrasses has been disregarded since its invaded areas were rather small and highly restricted (Winters et al., 2020). It is assumed that, due to habitat preferences, the competition of *H. stipulacea* with natives is neglectable (Sghaier and Zakhama-sraieb, 2011). Interestingly, a more recent study, on the eastern Tunisian coast (Sghaier et al., 2014), showed that *H. stipulacea* is replacing *Cymodocea nodosa*, which already disappeared where *H. stipulacea* reached its maximum invasion. A more recent introduction of this Red Sea native occurred in 2002 on the Caribbean Islands with completely different characteristics from those of the Mediterranean invasion (Ruiz and Ballantine, 2004). With first records dating from Grenada, *H. stipulacea* rapidly spread through the Caribbean Islands and already established along the coast of at least 19 islands (Willette et al., 2014). It has been found in monospecific patches or mixed with the native seagrasses *Thalassia testudinum* and *Syringodium filiforme* (Scheibling et al., 2018). Since only sterile or male plants have been spotted in the Caribbean, the rapid spread of this seagrass in the Caribbean Islands was exclusively attributed to the transfer of fragments by recreational boats (Vera et al., 2014; Willette et al., 2014). Additionally, its great acclimation capacity/habitat flexibility (Coppejans et al., 1992; Pereg et al., 1994), high tolerance to warm water temperatures, light intensities, nutrient levels, and salinities [reviewed by Gambi et al. (2009)], and the capacity to grow from the intertidal until depths up to 50 m (Beer and Waisel, 1982) might have further favored the invasion success (see Winters et al., 2020).

Although the negative influence of *Z. japonica* in the Northeast Pacific and of *H. stipulacea* in the Mediterranean on native relatives is debated, this is not the case in the Caribbean. *H. stipulacea* replaced entire meadows of the native manatee grass *S. filiforme* and *H. wrightii* (present at deeper locations) (Steiner and Willette, 2015) and its congeneric *Halophila decipiens* is in serious risk of local extinction.

Studying the genetic structure of *H. stipulacea* in the Caribbean Islands is needed to determine its similarity with the native (Red Sea, Persian Gulf, and Indian Ocean) and Mediterranean populations and understand their invasive behavior (Willette et al., 2014). Yet, the way organisms develop, interact with the surrounding, adapt to new environments, and ultimately survive and evolve strongly depend on the complex microbial communities they host (Rosenberg and Zilber-Rosenberg, 2018). As such, the individual phenotypes are the sum and/or combination of the host and associated microbial gene expressions (Zilber-Rosenberg and Rosenberg, 2008). So, to understand the invasive behavior and the success of some invasive species, we argue that one should also consider the usually underlooked associated bacterial community. In the light of the hologenome theory of evolution, in which all organisms harboring abundant and diverse microbiota are considered holobionts and act as a unit of selection in evolution (Zilber-Rosenberg and Rosenberg, 2008), the possible bacterial role in key functions that are part of the invasion stages (i.e., colonization, establishment, and spread) is potentially as important as studying the host physiology.

This new integrative approach has already been applied to terrestrial environments, showing that the rhizosphere-associated microbial communities can directly or indirectly facilitate the invasion process [see review by Coats and Rumpho (2014)]. In marine environments, the study of the microbiome of one of the most famous invasive seaweeds, *Caulerpa taxifolia*, has shown that the associated microbial community is stable enough to allow tracing the origin of the invasion (Arnaud-Haond et al., 2017). The same was shown for the invasive con-specific *Caulerpa cylindracea* (Aires et al., 2013) in the Mediterranean Sea. Some endophytic bacteria, showing a tight association with invasive *Caulerpa* species (Aires et al., 2013; Arnaud-Haond et al., 2017) and described as nitrogen-fixing and plant/algae growth enhancers, may have a role in *Caulerpa* success as an invader (Reinhart and Callaway, 2006). Moreover, *C. taxifolia* can change the sediment bacterial community composition and, consequently, its chemistry, inhibiting the growth of native seagrasses (Chisholm and Moulin, 2003; Gribben et al., 2017). The associated bacteria of another invasive genus, *Asparagopsis* sp., can adapt to the environment by shifting/acquiring a bacterial community that fits its needs (Aires et al., 2016).

Some studies on seagrasses have already provided evidence of direct links between seagrass physiological functions and their associated microbiome [see review by Ugarelli et al. (2017) and Tarquinio et al. (2019)]. This is further supported by the detection of a distinct microbiome in the seagrass phyllosphere (leaves) and rhizosphere (roots) (Ugarelli et al., 2018; Zhang et al., 2019). As different metabolic and functional processes dominate in these plant “microenvironments,” different bacterial members are specifically associated with these organs (Ugarelli et al., 2018). However, fewer studies compared the bacterial communities of different seagrass species and the few doing it are contradictory with some claiming species specificity of the microbiome while other did not find inter-specific differences (e.g., Martin et al., 2018; Ugarelli et al., 2018). This questions the importance of host phylogeny in shaping the bacterial community structure. None of these studies have ever compared invasive seagrass microbiomes with their native relatives. In the light of the hologenome theory, filling that gap would help not only to understand how some species can successfully thrive in a completely new environment, but it can also give us more tools to predict and/or eventually stop an invasion (Kowalski et al., 2015).

Here, we aimed to compare the bacterial communities of invasive and native seagrass species. We investigated, for the first time, the differences among the bacterial communities associated with two Caribbean native seagrass species *T. testudinum* and *H. wrightii* and the invasive seagrass species *H. stipulacea*, from six different locations on the island of Curaçao (Lesser Antilles). The associated bacterial community, inferred from 16S rRNA gene amplicon, was analyzed separately for leaves and roots to pinpoint their functional profile more specifically, given their ecophysiological differences. Our specific goals were (i) to assess whether bacterial community diversity and composition associated with seagrasses differs between native and invasive seagrass species, (ii) to show if the native-invasive differentiation holds for above- and below-ground tissues, and (iii) to infer the possible role of those bacteria in manufacturer’s protocol for “Solid Tissue Samples” using functional inference (Tax4Fun) and literature research. We hypothesized that bacterial communities would follow a species-specific pattern, but the invasive species would have a more distinct community compared to native seagrasses since it recently invaded from a different environment. Finally, we expected to find a community richer in bacteria with particular features [e.g., plant growth-promoting (PGP) factors] associated with the invasive species that would favor it when compared to the native species. Likewise, we also hypothesized that the invader *H. stipulacea* would show a higher proportion of putative functions that could also bring an advantage, by favoring plant growth, for the invasive plant over the native ones.

## Materials and Methods

### Sampling

**FIGURE 1:**
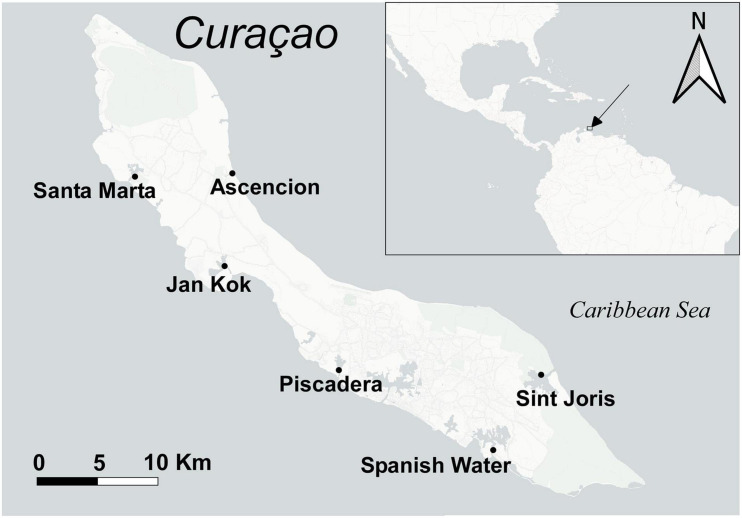
Sampling locations for the seagrasses around the Island of Curaçao. Sampling of each seagrass species was distributed as follows (with three replicates each for roots and leaves separately): Santa Marta—*H. stipulacea* + two seawater and three sediment samples; Jan Kok—*T. testudinum*, *H. wrightii*, and *H. stipulacea* + three seawater and three sediment samples; Piscadera—*T. testudinum* and *H. stipulacea* + two seawater and three sediment samples; Spanish Water—*T. testudinum*, *H. wrightii*, and *H.* stipulacea + four seawater and four sediment samples; Sint Joris—*T. testudinum* and *H. stipulacea* + one seawater and three sediment samples; Ascencion—*T. testudinum* and *H. wrightii* + two seawater and four sediment samples.

Samples were collected from six different locations around the island of Curaçao (Caribbean Sea) (Figure 1) in January 2017 with an average seawater temperature of 28–32°C. Three seagrass species were sampled: the native *T. testudinum* and *H. wrightii* and the invasive *H. stipulacea.* At each location, three replicate specimens of the species present in each site were collected along with environmental samples (i.e., sediment and seawater), resulting in a total of 112 samples (see the description in Figure 1). All seagrasses were collected in the shallow subtidal between depths of centimeters to a few decimeters below sea surface with a few meters distance between samples. The different species were mostly collected from monospecific meadows, though different species were always sampled close to each other with distances between meadows within 10 m. Only at the entrance of Spanish Water did the species occur in a sympatric meadow. Individual specimens were uprooted and washed free of sediment, epiphytes, and anything loosely attached using seawater from the sampling location. Excess seawater was shaken off and leaves and roots were separated and directly preserved in Xpedition^TM^ Lysis/Stabilization Solution (Zymo Research, California) and preserved at −20°C until further extraction. Collected sediment samples had a volume of roughly 1 ml and represented the sediment at the root depth of the respective seagrass. The portion of sediment, for each sample, was scooped using sterilized 2.5-ml tubes. Seawater bacterial communities were collected by filtering 0.5 L of seawater from the water column above the seagrass meadow, using 0.2-μm filters. The filters were preserved similarly to seagrasses and sediment samples. Environmental samples were collected as controls (1) to compare their bacterial community with those from the seagrasses and (2) to discard environmentally related operational taxonomic units (OTUs) when determining seagrass core OTUs.

### DNA Extraction and 16S Amplicon Sequencing

Total genomic DNA was extracted from all samples using the Quick–gDNA kit (Zymo Research^TM^) according to the manufacturer’s protocol for “Solid Tissue Samples” (page 4 of the manual). For all the samples, including sediment (0.25 g) and seawater filters, in the lysis step, we have used tungsten beads and an automatic homogenizer (for 2 min, at a frequency of 20/s) for a more efficient mechanic lysis. For bacterial community characterization, the nearly complete 16S rRNA genes were amplified using the universal primers 27F and 1492R with the following changes to the original protocol (Lane, 1991): initial denaturation at 95°C for 2 min, 35 cycles of denaturation at 95°C for 20 s, annealing at 55°C for 20 s, and extension at 72°C for 90 s, with a final extension at 72°C for 3 min. The 25-μl reaction mixture contained 250 μM dNTPs, 0.6 μM of each primer, 1 × 2 PCR buffer mix, 2 μl of template DNA (samples were diluted to a final concentration of about 10 ng μl^–1^ per reaction), and 0.3 μl of Taq polymerase (Advantage^®^ R2 Clontech^TM^). PCR products were cleaned using the ExoFastAP enzyme following the protocol of the manufacturer Thermo Scientific^TM^. Amplified DNA was sent to Molecular Research (MR DNA, Shallowater, TX, United States) where a nested PCR was performed prior to sequencing. The modified 8-bp key-tagged primer 799F along with the reverse primer 1193r, covering the regions V5–V7 of the 16S rRNA and amplifying a fragment of ∼400 bp, were used to avoid chloroplast cross-amplification (Bodenhausen et al., 2013). PCR conditions were as follows: 95°C for 3 min, 10 cycles of 95°C for 20 s, 50°C for 30 s, 72°C for 30 s, and a final elongation of 72°C for 3 min. The samples were pooled in equal proportions based on their molecular weight (calculated based on the size of the amplicon) and DNA concentrations (using Qubit Invitrogen^®^) and purified using calibrated Agencourt^®^ AMPure^®^ XP beads. DNA libraries were prepared by following Illumina TruSeq DNA library preparation protocol and paired-end (2 bp × 250 bp) sequencing performed at MR DNA^1^ (Shallowater, TX, United States) on an Illumina MiSeq system following the manufacturer’s guidelines.

The Q25 sequence data derived from the sequencing were processed using the MR DNA ribosomal and functional gene analysis pipeline (see Text Footnote 1, MR DNA, Shallowater, TX, United States). Sequences were depleted of primers; short sequences <150 bp and sequences with ambiguous base calls were removed. Sequences were quality filtered using a maximum expected error threshold of 1.0 and dereplicated. The dereplicated or unique sequences were denoised; unique sequences identified with sequencing or PCR point errors were removed, followed by chimera removal. The demultiplexing step was performed using the “split_libraries.py” Qiime command and default parameters. Subsequent data processing was done using QIIME version 1.9. (Caporaso et al., 2010) and clustered into OTUs at >97% similarity using open-reference OTU picking with the UCLUST algorithm against the SILVA reference database (version 132^2^). After selection of one representative sequence per OTU, the database was aligned. Taxonomy was assigned using the SILVA database (version 132). From the resulting OTU table, eukaryotic organelle sequences (i.e., chloroplasts and mitochondria) and unassigned sequences were removed, as well as rare OTUs (singletons and doubletons). The table used for downstream analysis is from now on referred to as OTU table.

### Seagrass-Associated Bacterial Communities

One of the samples (root from *H. wrightii* sampled in Ascension) resulted in a very low number of sequences (7,217), compared to the remaining 111 samples (median: 65,124), likely due to DNA extraction, PCR, or sequencing problems. Hence, we decided to omit it from our final dataset. For statistical purposes, samples were normalized by rarefaction to the minimum number of sequences (29,021), per sample, and used in alpha and beta diversity analysis. Rarefaction was performed to adjust for differences in library sizes across samples to aid comparisons of alpha and beta diversity. The rarefaction method chosen (rarefaction to the minimum number of sequences) allows comparability with previous studies (using that same method). For all the other compositional analysis (differential analyses and core community), non-rarefied data were used so we would have the real proportion of the OTUs/taxa analyzed without the “randomly selected” factor of the rarefaction method.

#### Bacterial Community Richness and Diversity—α-Diversity

Bacterial richness was estimated using the number of OTUs, and the Shannon index was used to include evenness, as calculated in QIIME using the alpha_diversy.py script. The Shannon diversity index was exponentiated to reflect the true diversity. Boxplots were generated for a visualization of the α-diversity differences among groups of samples (pooled replicates according to species and tissue type). Data were tested for normal distribution with the Shapiro–Wilk test in the software program R (version 3.4.2). As data were not normally distributed, and to maximize comparability with the analysis of β-diversity, univariate PERMANOVA was used to determine significant differences (as in Hartmann et al., 2015) among species, locations, and type of tissues (described in detail below). Matrices were constructed using Euclidean distance, and the number of permutations was set to 999. Monte Carlo tests were done when the number of unique permutations was less than 900 (Metropolis and Ulam, 1949).

#### Bacterial Community Composition and Structure—β-Diversity

For comparative purposes, the bacterial community composition and structure were assessed through OTU presence/absence and square root transformations, after which a resemblance matrix was compiled using the Bray–Curtis dissimilarity algorithm. Statistical analyses were done using a three-factor PERMANOVA: species (fixed factor with three levels), tissue type (fixed factor with two levels), and location (random factor with six levels). All the factors and respective interactions were tested. Monte Carlo test was done when the number of unique permutations was less than 900. Both results had the same output; hence, downstream analysis only considered the results for square root transformation method. The deviation of the centroid was calculated using PERMDISP to detect differences among variances of the sample groups. To assess the differentiation among the seagrasses and the environmental samples (sediment and seawater), a one-way PERMANOVA test for the factor “tissue type” (four levels—root, leaf, sediment, and seawater) was performed.

Community differentiations were visualized with Canonical Analysis of Principal coordinates (CAP), which is a better match for *a priori* hypothesis testing plots using the interaction of species and tissue type (Species × Tissue type) as *a priori* factor. CAP also visualized the statistical differentiation among environmental and seagrasses samples, seawater, and sediment. A separated Principal Coordinate Analysis (PCoA) was done for roots and leaves separately, and discriminant vectors (with a Pearson correlation > 0.6) were overlaid for characterization of the taxa discriminating the multivariate patterns. Because not all OTUs were assigned to the same taxonomic level (some would go to the genus level some just to the order level), the vectors show different levels of taxonomic characterization.

*Halophila stipulacea* is known for its tolerance/resistance to a wide range of salinities (including the hypersaline waters of the Suez channel) compared to other seagrasses. Most seagrass species are adapted to grow at salinities ranging from 20 to 40 Practical Salinity Units (PSU) (Touchette, 2007) while *H. stipulacea* has been shown to thrive in a range of 25–60 PSUs (Oscar et al., 2018). This feature has been associated with *H. stipulacea* invasive success and advantage over other sympatric seagrasses [see review by Winters et al. (2020)]. The reason for this higher tolerance is not deeply studied, but it was suggested that the associated microorganisms could have a role (Touchette, 2007). Based on a review featuring several halotolerant bacteria associated with halophytes (Etesami and Beattie, 2018), we have selected and filtered, from our OTU table, the bacterial genus depicted in Table 1 from the previously mentioned review. Those were used to construct a bar chart showing the different proportions of each bacterial genus in each seagrass species. The considered bacterial genera, besides being halotolerant/halophilic, are also known for its PGP capabilities and used to stimulate plant growth and increase the tolerance of non-halophilic crops to salinity (Etesami and Beattie, 2018). The relative abundances (calculated for each seagrass species by dividing the number of reads of each halotolerant bacterium by the total number of reads in that species) were compared in the three different species to investigate their possible role in the adaptation of the invasive *H. stipulacea* to new environments and consequent invasiveness. PERMANOVA tests were performed to assess the differences between halophilic/halotolerant bacteria abundances in the three different species and across tissues.

All the statistical analyses in this manuscript were performed using PRIMER-E + PERMANOVA v.6 (Clarke and Gorley, 2006). The statistical tests performed in this study were carried out using an OTU table evenly rarefied to the minimum number of 29,021 sequences (per sample) and considered significant at *p* < 0.05.

In this study, when referring to the microbiome, we mean bacterial composition only (associated bacterial community).

#### Differential Analysis of Bacterial Communities

To further examine the distinction between the native and invasive microbiomes, differential analyses (Deseq function in the Deseq2 package in R) were done using the bacterial reads taxonomically assigned up to genus level. The analysis used a negative binomial distribution to model the read count data, which accounts for the over-dispersion (variance > mean) of the data (Love et al., 2014). Deseq2’s method median-of-ratios was used to normalize the count data (Anders and Huber, 2010). To test for significant differences among the seagrass species, the Wald statistics, with an alpha of 0.05, was used. The *p*-values attained by the Wald test were corrected for multiple testing using the Benjamini and Hochberg method to reduce the possibility of false positives [adjusted *p*-values (Benjamini and Hochberg, 1995)]. The method excluded genera with a low number of reads and outlier samples. *p*-values that did not pass the filtering method are outputted as NA values. To visualize the differential read abundances among the seagrass species, a variance stabilization transform was performed, from which a heatmap was created with the pheatmap command in the pheatmap package in R.

#### Seagrass Core Microbiome

The core microbiome of the three seagrass species (independently calculated for the three species) was determined using the non-rarefied OTU table, to assess the real proportion of the taxa analyzed. A bacterial OTU was considered as part of the “core” microbiome when it occurred in at least 80% of the samples of a certain species. Furthermore, all OTUs with a relative abundance above 0.01% in seawater or sediment were labeled as “environmental bacteria” and were removed from the initial seagrass core results. Established per species-cores were displayed in Venn diagrams to sort out the seagrasses core (common to all three species) and species-specific OTUs. Barplots were created to visualize the microbial taxa occurring in the core microbiomes. The core microbiome was determined in QIIME version 1.9 (Caporaso et al., 2010) using the compute_core_microbiome.py script.

### Prediction of Functional Profiles

In order to predict the functional profile of the seagrass-associated bacterial communities, a taxonomic profile of KEGG Orthology (KO) pathways was obtained from our SILVA-based OTUs (version 132) using the Tax4Fun2 library in R (Wemheuer et al., 2020). Within the Tax4Fun2 library, the functional prediction was obtained using the *runRefBlast* function with the database mode set to “Ref100NR” and the path_to_otus argument set to the OTU representative sequences fasta file. The function performed a next neighbor search against the 16S rRNA reference database using the BLAST algorithm. Subsequently, the *makeFunctionalPrediction* function was run. This function summarized the results of the next neighbor search performed during the *runRefBlast* in a relative abundance table of KO pathways per sample. The path_to_otu_table argument was set to the OTU table. The *min_identity_to_reference* argument was set to 0.95, meaning that OTUs passing a similarity threshold of 95% to their next neighbor were considered for further analysis. The specified cutoff level results in predicted function for a given OTU at an approximately generic level. This level was chosen as metabolic capabilities among prokaryotes have shown to vary also below family level (Goldford et al., 2018).

#### Differential Analysis of the Predicted Functional Profiles

A differential analysis (Deseq function in Deseq2 package in R) was used to further examine host species differentiation by tissue in the putative functional profile of the associated bacterial community based on the relative abundance table of KEGG orthologs. The analysis and adjacent statistics were performed using the same method as for the bacterial read count differential analysis (see Section “Differential Analysis of Bacterial Communities”) (Love et al., 2014).

## Results

The resulting raw dataset consisted of a total of 9,472,379 sequences. After quality control, and removal of chimera sequences, chloroplast sequences, unassigned sequences, and “singletons and doubletons,” a total of 8,533,147 high-quality sequences were retained for analysis (mgm4868291.3 – mgm4868401.3^3^), resulting in a total of 257,773 unique OTUs.

### Bacterial Community Richness and Diversity—α-Diversity

The calculated diversity indices (Observed OTUs and exponentiated Shannon diversity—the order in which the *p*-values are presented below) showed differences among seagrass species (*F*_2,73_ = 6.904, *p* = 0.002/*F*_2,73_ = 4.987, 0.003) and tissues (*F*_1,73_ = 92.487, *p* = 0.001/*F*_1,73_ = 49.072, 0.004). Only the Observed OTUs index showed differences among locations, which depended on the seagrass species and tissue (Species × Location *F*_5,73_ = 2.22, *p* = 0.034; Tissue × Location *F*_5,73_ = 2.985, *p* = 0.017).

**FIGURE 2:**
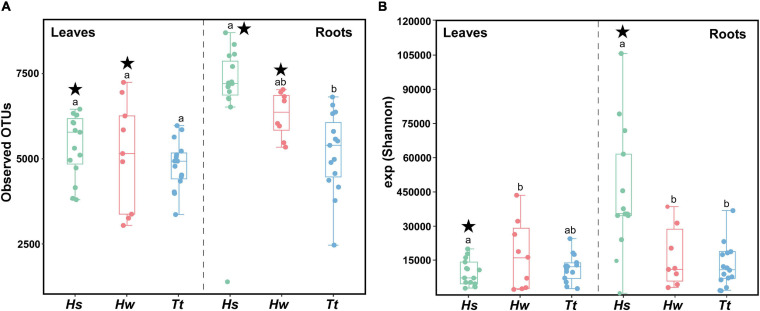
Boxplot for the bacterial α-diversity using **(A)** observed OTUs and **(B)** exponentiated Shannon Diversity Indices of bacterial communities associated with the different tissues of the three seagrass species based on Illumina 16S rRNA gene amplicon sequencing. Hs—*Halophila stipulacea*, Hw—*Halodule wrightii*, Tt—*Thalassia testudinum*. The line within the box represents the median, and the bottom and top boundaries of the box represent the 25th and 75th percentiles, respectively. The whiskers represent the lowest and highest values within the 1.5 interquartile range (IQR). Different lowercase letters indicate significant differences in mean value for species within tissues at *p* < 0.05. Stars mark significant differentiation of tissues within species at *p* < 0.05. Graphics were manipulated to both fit in the same figure. In addition, colors were changed so the different species’ colors would match with the other figures.

Differences among species depend on the tissue analyzed for both indexes (Species × Tissue *F*_2,73_ = 20.933, *p* = 0.001/*F*_2,73_ = 25.318, 0.001). Looking at the observed OTUs index, the invasive *H. stipulacea* only shows a higher diversity when comparing their root community with that of *T. testudinum* (Supplementary Table 5 and Figure 2). However, the exponentiated Shannon results show that the invasive roots’ community diversity is higher than both native species (Supplementary Table 13 and Figure 2), while for the leaves, *H. wrightii* bacterial diversity is higher than that of *H. stipulacea* (Supplementary Table 13 and Figure 2). All the *p*-values for the α-diversity statistical analysis are in Supplementary Tables 1–13.

### Bacterial Community Composition and Structure—β-Diversity

**FIGURE 3:**
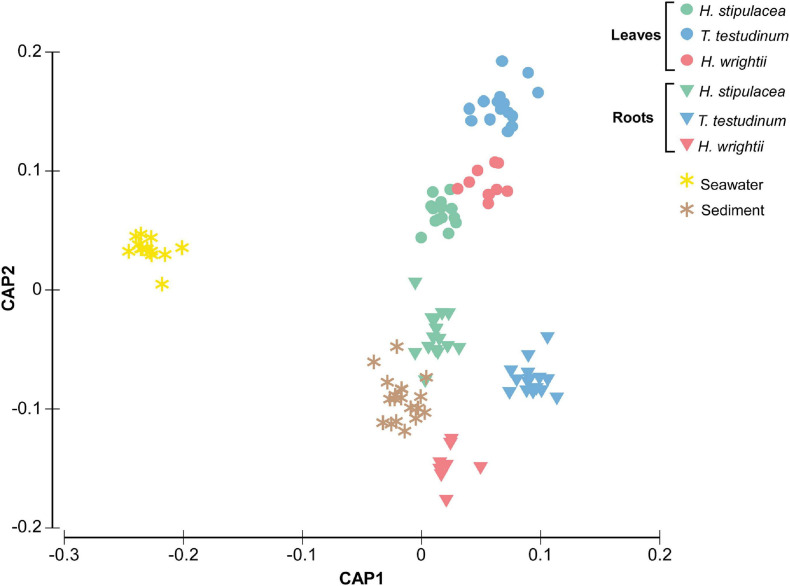
Canonical analysis of principal coordinates (CAP) ordination plot (based on Bray–Curtis dissimilarity matrix of square root transformed bacterial abundances) showing canonical axes that best discriminate the bacterial community assemblages of the native seagrasses *H. wrightii* and *T. testudinum* and the invasive *H. stipulacea* across leaves and roots tissues as well as seawater and sediment in six bays of the Caribbean island of Curaçao. Colors were changed so the different species’ colors would match with the other figures.

Multivariate analysis disclosed a clear separation of bacterial communities for the different factors (species, tissue, and location) and their interactions (Table 1 and Figure 3). Environmental bacterial community structure from sediment and seawater differed strongly from each other (*p* = 0.001, Figure 3) as well as from the seagrass-associated bacterial communities (*p* = 0.001, Figure 3). Within the seagrasses, there was a strong composition differentiation between roots and leaves (Table 1 and Figure 3), explaining about 11% of the total variation. In addition, the bacterial community structure differed among the three seagrass species (Table 1, Figure 3, and Supplementary Table 14), explaining close to 7% of the total variation. The interaction of Tissue and Species explained another 11% of the variation. So, seagrass species and tissue formed the most important structuring factors of the seagrass microbiome composition. As a random factor, location contributed little to the total variation (∼3%). Pairwise tests showed differentiation among all locations within all three seagrasses (*p* = [0.001–0.027]).

**TABLE 1:** PERMANOVA main test for the structure of the bacterial community associated with the native seagrasses *Halodule wrightii* and *Thalassia testudinum* and the invasive *Halophila stipulacea* across leaves and roots tissues in six bays of the Caribbean island of Curaçao.

Source of variation	Num.df	Den.df	Pseudo-*F*	*p* (*perm*)^1^	PERMDISP (*p*)	Variation explained
Species	3	73	4.156	**0.001**	0.062	6.6%
Tissue	1	73	10.587	**0.001**	0.948	11.2%
Location	5	73	2.125	**0.001**	0.409	3.4%
Species × tissue	3	73	3.667	**0.001**	**0.001**	11.1%
Species × location	10	73	1.562	**0.001**	0.929	5.0%
Tissue × location	5	73	1.624	**0.001**	0.147	3.8%
Species × tissue × location	10	73	1.466	**0.001**	0.326	8.3%

*^1^PERMANOVA values were calculated after square root transformation and Bray–Curtis distances calculation for OTU composition. H0: there are no differences in the distribution of OTUs for the different treatments, H_0_ rejected if *p* < 0.05. Boldface values are significant at *p* < 0.05, *p*-values based on 999 permutations. PERMDISP H_0_: there are no differences in dispersions among *a priori* groups. H_0_ rejected if: *p* < 0.05.*

Differentiation among microbiomes of all three seagrass species was detected in leaves and roots (Supplementary Table 17) and within every sampled bay (Supplementary Table 18). All the *p*-values for the β-diversity statistical analysis that are not represented in Table 1 are in Supplementary Tables 14–24.

**FIGURE 4:**
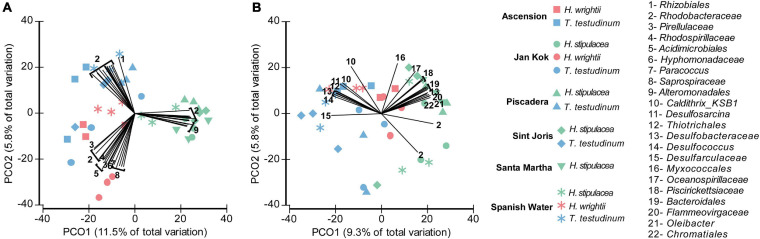
Principal coordinate analysis (PCO) plot (based on Bray–Curtis dissimilarity matrix of square root transformed bacterial abundances) showing canonical axes that best discriminate the bacterial community assemblages of leaves **(A)** and roots **(B)** across the three seagrass species (*H. stipulacea*—green, *H. wrightii*—red, and *T. testudinum*—blue) and locations (Ascension—square, Jan Kok—circle, Piscadera—triangle, Sint Joris—diamond, Santa Marta—inverted triangle, and Spanish Water—asterisk). Displayed vectors show the bacterial groups with a Pearson correlation larger than 0.6. Colors were changed so the different species’ colors would match with the other figures.

In the leaves, the invasive *H. stipulacea* microbiome differentiation from the two native seagrasses correlated strongly positive with the read abundance of *Alteromonadales* (Figure 4A, vectors 9) and specific OTUs from the *Rhodobacteraceae* family (Figure 4A, vectors 2). The family *Rhodobacteraceae* had specific members in the leaves of each of the three seagrasses, but especially in *T. testudinum*. *T. testudinum* microbiome structure also showed a distinguished correlation with members of the *Rhizobiales* (Figure 4A). The bacterial groups driving root differentiation among seagrass species were very different and more diverse. Members of, for example, the families *Rhodobacteraceae, Piscirickettsiaceae*, and *Oceanospirillaceae* and the orders *Chromatiales* and *Bacteroidales* positively correlated with the invasive *H. stipulacea* and members of the *Caldithrix* are more associated with native seagrasses, especially *T. testudinum* (Figure 4B). Some members of *Desulfobacteraceae* were rather specific for the invasive seagrass root while others were so for the native seagrass roots (Figure 4B). Native seagrass roots showed a higher number of discriminating taxa vectors of sulfate-reducing bacteria (e.g., *Desulfosarcina*, *Desulfobacteraceae*, etc.) (Figure 4B).

#### Core- and Species-Specific Community Members

**FIGURE 5:**
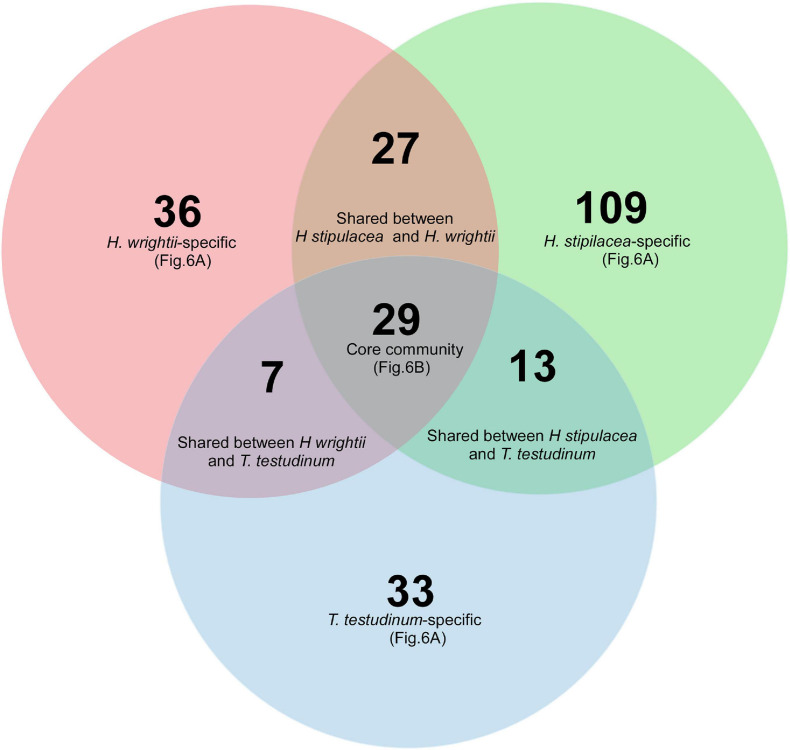
Venn diagram representing core bacterial OTUs occurring in at least 80% of the seagrass samples of *H. stipulacea* (green), *H. wrightii* (red), and *T. testudinum* (blue) in both roots and leaves and the different intersections among them. OTUs specific to each seagrass species (species-specific) and OTUs common to all three seagrasses (“core of the core” community) are represented in Figure 6 at its order level.

Compared to the total number of OTUs, analysis of the core community showed a low number of OTUs in a tight association (present in more than 80% of the samples) with each of the seagrasses. Out of the 254 selected OTUs (occurring in at least 80% of the samples from a certain species and with a relative abundance inferior to 0.01% in seawater or sediment), only 29 OTUs were shared among all three seagrass species (“core of the core” OTUs). The invasive *H. stipulacea* showed approximately three times more “species-specific” OTUs (109) than the native seagrasses: *H. wrightii* (36) and *T. testudinum* (33) (Figure 5), probably as a reflection of the higher α-diversity. The native seagrass species shared less core OTUs (3.9%) than between natives and the invasive *H. stipulacea* (5% with *T. testudinum* and 9.7% with *H. wrightii*; Figure 5).

**FIGURE 6:**
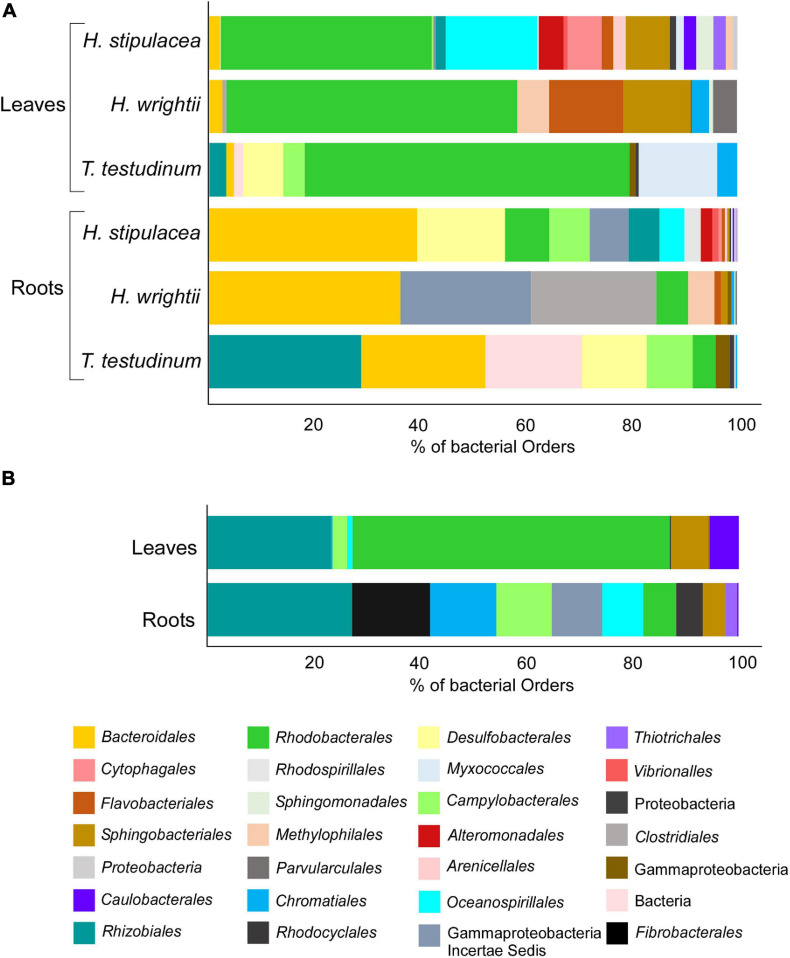
Seagrass core species-specific bacterial structure based on the Venn diagram from Figure 5 with OTUs grouped by order level: **(A)** Roots and leaves of *H. stipulacea*, *H. wrightii*, and *T. testudinum* and **(B)** their shared core community (“core of the core”), associated with roots and leaves. Colors were changed so the same bacterial order would have matching colors in the different graphs.

That number of OTUs translated into a distinct distribution through different bacterial orders (Figure 6—that taxonomic level was chosen in order to have a clearer graphic). *H. stipulacea* displays 21 “species-specific” orders and *H. wrightii* and *T. testudinum* species-specific OTUs resulted in 11 and 10 orders, respectively (Figure 6A). The three seagrasses shared 11 Orders distributed between the two different tissues (Figure 6B). The OTUs found to be “species-specific” for each of the three species (Figure 5) were mostly in the same bacterial orders (Figure 6). For all three seagrasses, *Rhodobacterales* was the most common core order associated with leaves, followed by *Oceanospirillales* for *H. stipulacea*, *Flavobacteriales* for *H. wrightii*, and *Myxococcales* for *T. testudinum* (Figure 6A). However, only *H. stipulacea* and *H. wrightii* shared the most common root core order, *Bacteroidales* (Figure 6A), which was the second most abundant for *T. testudinum* following *Rhizobiales* (Figure 6A). Gammaproteobacteria and *Desulfobacterales* were the second most abundant root core orders in *H. stipulacea* and *H*. *wrightii*, respectively (Figure 6A).

#### Differential Analysis of Bacterial Communities

**FIGURE 7:**
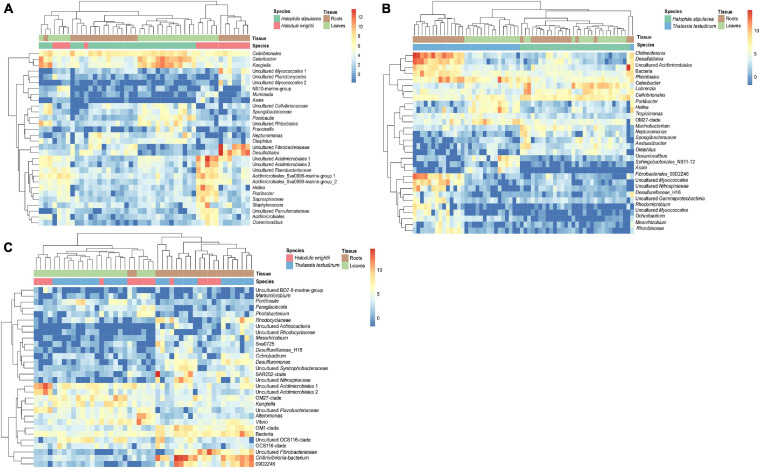
Heatmaps of the seagrass species-associated bacterial community differential analysis. Displayed are the *H. stipulacea* vs. *T. testudinum* **(A)**, *H. stipulacea* vs. *H. wrightii* **(B)**, and *H. wrightii* vs. *T. testudinum* **(C)** variance stabilized transformed bacterial counts of the 30 most different abundant genera (lowest *p*-values, *p* < 0.05). Samples were ordered and clustered based on their Euclidean distance. Taxonomic names are displayed up to order level.

We detected more differentially abundant bacterial genera between the invasive and native seagrass species (*H. stipulacea* vs. *T. testudinum* = 312, *H. stipulacea* vs. *H. wrightii* = 138) than between native species (*T. testudinum* vs. *H. wrightii* = 97). Relative to *H. stipulacea*, 77% of the differential abundant genera showed increased abundance in *T. testudinum*, and 63% showed increased abundance in *H. wrightii*. Relative to *H. wrightii*, 68% of the differential abundant genera showed increased abundance in *T. testudinum*. Among the 30 most differential abundant bacterial genera, members of the genus *Desulfatitalea*, two genera of *Myxococcales*, and several genera of *Fibrobacteres* revealed significantly higher abundances in the roots of the native seagrasses compared to the invasive species *H. stipulacea* (Figures 7A,B, *p*-values: *p* < 0.05E-5, Supplementary Tables 25, 31). *Oceaniovalibus* sp. and *Asaia* sp. showed elevated read abundance in leaf samples of *T. testudinum* and *H. wrightii* relative to *H. stipulacea* (Figures 7A,B, *p*-values: *p* < 0.05E-3, Supplementary Tables 30, 31). *Celeribacter* revealed overall (in leaves and roots) higher abundance in *H. stipulacea* relative to the native species (Figures 7A,B, *H. stipulacea* vs. *T. testudinum*—*p* = 1.98E-19 and *H. stipulacea* vs. *H. wrightii*—*p* = 2.91E-17, Supplementary Tables 30, 31). The differential analysis comparing both native species (*T. testudinum* and *H. wrightii*) showed a more conspicuous separation by tissue than by species (Figure 7C). *Fibrobacteres* showed relative higher abundances in the roots of both *T. testudinum* (*p* = 6.48E-16 and *p* = 1.91E-13, Figure 7C, Supplementary Table 32) and *H. wrightii* (*p* = 5.97E-04, Figure 7C, Supplementary Table 32) although represented by different genera. Relatively higher abundances of *Desulfuromonas* (*p* = 8.66E-05, Figure 7C, Supplementary Table 32) and two unknown genera of *Rhodocyclaceae* (*p* = 9.41E-12, *p* = 8.22E-4, Figure 7C, Supplementary Table 32) were associated with *T. testudinum* roots, while the genus *Ponticaulis* showed to be differentially related to *T. testudinum* leaves. Also, two genera assigned to the order *Acidimicrobiales* showed relative higher abundances in several samples of *H. wrightii* leaves (*p* = 2.35E-07, *p* = 1.79E-04, Figure 7C, Supplementary Table 32), whereas another *Acidimicrobiales* showed relative higher abundances in the roots of *T. testudinum* (*p* = 2.88E-04, Figure 7C, Supplementary Table 32).

#### Relative Abundance of Halophilic Bacteria

**FIGURE 8:**
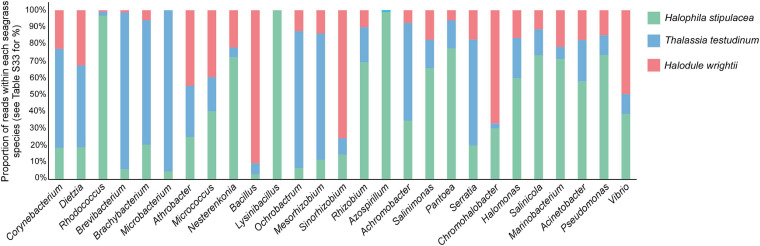
Relative abundance of potentially halophilic/halotolerant bacteria (according to Etesami and Beattie, 2018) associated to each of the seagrass species sampled in all the locations and independently of the tissue. Proportions were calculated, for each species, by dividing the number of reads of each halotolerant bacteria by the total number of reads in that species. Due to the low value and high discrepancy among proportions for each halotolerant genus, a regular bar chart would not allow the visualization of all the bars for all the genera; therefore, we used a 100% stacked column chart. The graph is displaying the relative composition of halophilic/halotolerant bacteria (according to Etesami and Beattie, 2018), which represents less than 2% of the overall associated bacterial community. Real proportions are presented in Supplementary Table 28.

Out of the 27 halophilic/halotolerant genera, listed in Etesami and Beattie (2018) and found in our OTU table, the relative abundance of reads is higher for 13 genera associated with the invasive *H. stipulacea*, 9 genera associated with *T. testudinum*, and 5 associated with *H. wrightii* (Figure 8 and Supplementary Table 28). The genera *Rhodococcus*, *Lysinibacillus*, and *Azospirillum* are highly represented in *H. stipulacea* compared to the native species (*Rhodococcus* is more than 40 times more frequent in *H. stipulacea*, *Lysinibacillus* is absent in the native species, and *Azospirillum* is more than 100 times more frequent in *Halophila* and absent in *Halodule*). More importantly, PERMANOVA tests showed that differences among species were significant (*H. stipulacea* vs. *T. testudinum*—*p* = 0.001, *H. stipulacea* vs. *H. wrightii*—*p* = 0.001, *T. testudinum* vs. *H. wrightii*—*p* = 0.007). Moreover, when looking at the tissue differentiation, we can see that this trend only holds for the leaves (Hs vs. Tt—*p* = 0.001 and Hs vs. Hw—*p* = 0.001, Tt vs. Hw—*p* = 0.035, Supplementary Figure 1A) while for the roots, native species did not show significantly different abundances of halophilic/halotolerant bacteria (*p* = 0.056) and only *H. stipulacea* roots tested significantly different from both of the native roots (Hs vs. Tt—*p* = 0.001 and Hs vs. Hw—*p* = 0.003, Supplementary Figure 1B).

### Prediction of Functional Profiles

Using Tax4Fun2, a table with the predicted gene copy numbers was created displaying a total of 365 KEGG Orthologies and their associated level 1, level 2, and level 3 pathways. The total amount of OTUs used in the prediction was 17,887, whereas the proportion of sequences not used in the functional prediction varied among samples from 0.35 to 0.91 (Supplementary Tables 29–31).

#### Differential Analysis of the Predicted Functional Profiles

The relative abundance of predicted gene copy numbers differed significantly among several KEGG orthologs between the native and invasive seagrass species, whereas the predicted functional profile of *H. wrightii* and *T. testudinum* showed almost none significant differences (summarized in Table 2 and Supplementary Tables 32–36). Most profound, the roots of the invasive *H. stipulacea* were predicted to be enriched in pathways related to “Lipoic acid metabolism,” “Glycine, serine, and threonine metabolism,” and “Taurine and hypo taurine metabolism” compared to the native species *H. wrightii* and *T. testudinum* (*p* < 0.05, Supplementary Tables 32, 33). ko00920, involved in Sulfur metabolism, showed to be well represented among all species but was slightly increased in *H. wrightii* roots compared to *H. stipulacea* (*p* = 0.034, Supplementary Table 33), and Ko00910, involved in Nitrogen metabolism, was predicted to be significantly enriched in both native species’ roots as opposed to *H. stipulacea* (*p* < 0.05, Supplementary Tables 32, 33). In the leaves, *H. stipulacea* showed an enrichment of several KEGG orthologs related to Carbohydrate digestion and absorption, biosynthesis of staurosporine, and biosynthesis of Tetracycline in contrast to the native species’ leaves (*p* < 0.05, Supplementary Tables 34, 35), whereas functional pathways related to Alanine, Aspartate, and glutamate metabolism and the biosynthesis of Arginine were predicted to be enriched in the native species’ leaves in contrast to *H. stipulacea* (*p* < 0.05, Supplementary Tables 34, 35).

**TABLE 2:** Total amount of KEGG orthologs with significantly different (*p* < 0.05) predicted gene copy numbers in the roots and leaves of the three seagrasses *Halophila stipulacea*, *Halodule wrightii*, and *Thalassia testudinum*.

Seagrasses comparison	Leaves	Roots
*H. stipulacea, T. testudinum*	50	104
*H. stipulacea, H. wrightii*	37	76
*T. testudinum, H. wrightii*	2	1

## Discussion

In contrast with terrestrial plants, the success of invasive seagrasses has not previously been investigated in a holobiont perspective. This is, therefore, a pioneer comparison of the bacterial communities of an invasive seagrass species (*H. stipulacea*) with the two natives (*H. wrightii* and *T. testudinum*) that it displaces, across the Caribbean. Increasing the current knowledge on the invasive species by studying the microbiome component, our holistic approach showed the potential to provide a broader perspective and increase the current understanding of the invasion process. By evaluating and comparing the bacterial community of the threatening, invasive species with those of the threatened native ones, we were able to assess diversity differences and specific bacterial taxa and putative function, which could be linked to the success of the invasion. As a pioneer, and by identifying possible bacterial taxa and/or invasive vs. native key differences in the bacterial community, this study sheds light and opens a path for a deeper investigation on the holobiont components that could be implied in the success of seagrass invasions in particular and plant invasions in general. Below, we discuss our findings on diversity, species-specific, shared, and core bacterial community structures for the three seagrasses and use the distinct taxonomic patterns (linked or not to distinct putative functional profiles) as clues to help to understand the success of *H. stipulacea* in the Caribbean.

### α-Diversity of the Seagrass Microbiomes

The bacterial community associated with the roots of the invasive seagrass, *H. stipulacea*, was highly diverse (for both indexes used), and that diversity was higher when compared to those of the native species. During the process of introduction and invasion, introduced individuals usually go through a bottleneck that is thought to strongly reduce the genetic diversity of the introduced macro-organism and a stripping of associated organisms (Rocca et al., 2019). However, other studies have also reported a higher diversity of rhizosphere microbial communities of several terrestrial plants in the invasive compared to their native range (Coats and Rumpho, 2014; Dawson and Schrama, 2016; van der Putten et al., 2016; Lu-Irving et al., 2019). Given the higher diversity of *H. stipulacea* compared to the two native species, we could assume that the invasive species (i) may profit from that habitat shift acquiring a more diverse bacterial community that would give it an advantage for a successful establishment and proliferation (e.g., by improved nutrient acquisition) or (ii) is naturally highly diverse and there was actually a decrease from its native range that was not enough to go as low as the diversity of the native species. These assumptions could only be tested by sampling *H. stipulacea* in its native habitat and comparing its α-diversity to that of in its invaded habitat. Previous results on bacterial communities associated with roots and leaves of *H. stipulacea* in the Red Sea (Mejia et al., 2016; Rotini et al., 2017) have shown a much lower α-diversity (Shannon) for leaves (4.05 ± 0.21–5.04 ± 0.15) and roots (4.49 ± 0.02–5.30 ± 0.46) when compared to our results (8.97 ± 0.64 and 10.35 ± 1.20, leaves and roots, respectively). This gives some strength to our first assumption. However, these authors have used 454 sequencing technology with sequencing depths much lower than those accessed by Illumina sequencing, as well as different primers, both compromising the direct comparison between ours and these two studies.

### Bacterial Community Structure and the Possible Role of Specific Bacteria

Increasing the knowledge on the different factors shaping the bacterial community of seagrasses and unveiling some key taxa associated to them would contribute to understand the influence of the microbiome on the invasion process, especially when co-analyzing invasive and native species. Bacterial community structure was seagrass species-specific for both root and leaf communities. So far, there are only a few studies that compare the bacterial communities of different seagrass species, and their results are contradictory. For example, Roth-schulze and Thomas (2016) studied the diversity of microbial communities on different types of host surfaces (including the seagrass species *Posidonia australis* and *H. ovalis*) and found that the bacterial community structure was unique to each host. Likewise, Martin et al. (2018) found that the microbial community structure of the rhizosphere of three co-occurring seagrass species, *H. ovalis*, *Halodule uninervis*, and *Cymodocea serrulata*, was unique for each species. In contrast, a recent study on the phyllo- and rhizosphere of the seagrasses *T. testudinum* and *S. filiforme* found that the microbial composition of roots and leaves was different without showing species specificity (Ugarelli et al., 2018). At a rhizosphere level, a lack of species specificity for bacterial communities but rather regional differences were reported for *Zostera noltii* and *Zostera marina* in Roscoff (France) and Faro (Portugal) (Cúcio et al., 2016). In our case, we detected species specificity at the island and tissue level, but not between co-occurring or neighboring seagrass species within bays. This can be due to our relatively low replication level at this scale. In contrast, seagrass-associated communities differed across all bays within each seagrass species indicating the importance of local effects. Overall, these results show that the composition of seagrass-associated bacterial communities is influenced by different factors such as host species, “microenvironment”/tissue (leaves and roots), and, ultimately, location.

As known from other studies that compared the microbiomes associated with the different plant tissues (e.g., Lebeis, 2015; Ettinger et al., 2017; Rotini et al., 2017; Ugarelli et al., 2018), the separation between leaves and roots was very clear for all the seagrass species in this study, with certain bacterial phylotypes (taxonomic groups) discriminating each of the tissues. This factor (tissue) was the one showing the highest explanation for bacterial community variation among seagrasses (11.2% for the overall differentiation). Representing the different tissues (roots and leaves) in different PCO plots shows the differentiation among the different species better (which was also statistically supported), especially at the leaf level where native seagrass species clearly segregated from the invasive *H. stipulacea*. For the leaves, the “invasive cluster” has 12 discriminant vectors assigned to the order *Alteromonadales*. This order was also found to be exclusively associated with *H. stipulacea* in our core community analysis (more than 50% of those OTUs were assigned to the genus *Alteromonas*), being present in both leaves and roots but in a higher abundance in the leaves. *Alteromonadales* was also found to be one of the most abundant bacterial orders associated with *H. stipulacea* from the northern part of the Red Sea in two independent studies (Mejia et al., 2016; Rotini et al., 2017). Also, Weidner et al. (2000) isolated a bacterium closely related to *Alteromonas macleodii* from *H. stipulacea* leaves, which is responsible for producing PGP oligosaccharides (Ferrier and Martin, 2002). These results provide support to the possible important and structural role that this phylotype may have for this seagrass species. Besides, some *Alteromonas* species isolated from algae have been described as having enzymatic activities relevant to the degradation of macroalgal cell walls (Goecke et al., 2010). Having those bacteria associated with their leaves could bring an advantage to this invasive species by helping it to get rid of the algal epiphytes and mitigate their possible competitive effect for light and gas exchanges that leads to reduced plant fitness (Brodersen et al., 2015). In addition, *Alteromonadales* have been suggested to collaborate with *Thiotrichales* to degrade marine dissolved organic matter in seawater (Mccarren et al., 2010). Our results on the core bacterial community analysis, specific to each species, show that *Thiotrichales* also show up exclusively associated with *H. stipulacea*, mainly and most abundantly with leaves, which could be a clue for that concerted work between these bacteria to bring some advantage to the invasive species. Experiments in axenic cultures of this seagrass species with subsequent inoculation of these two phylotypes could help clarify the correlation between these bacteria and its positive effect in *H. stipulacea*. However, obtaining axenic seagrasses is a challenge by itself.

In the native species, the leaf microbiome also forms statistically different clusters but with some level of mixing among some *T. testudinum* samples with the *H. wrightii* cluster. In that mixed cluster, we can find several discriminating vectors corresponding to the different differential bacteria, showing a lower level of specificity than that of the invasive species. The cluster that gathers most of the *Thalassia* samples shows the order *Rhizobiales* as a differential vector of this species that are known to fix nitrogen. Bacteria from the order *Rhizobiales* were consistently found in high relative abundances in root and leaf communities of phylogenetically diverse plant hosts and hence were considered part of the core plant microbiota (Garrido-Oter et al., 2018). The only vector that was common to all the seagrass species belonged to the family *Rhodobacteraceae*. Regardless, some of the OTUs assigned to the *Rhizobiales* order were found exclusively associated with *Thalassia* samples; our core analysis also shows this order as one of the most abundant orders common to all the three species. In other studies, these bacteria were found consistently and abundantly associated with leaves of several seagrass species as *H. stipulacea* (Mejia et al., 2016; Rotini et al., 2017), *Thalassia hemprichii* (Ling et al., 2015), *T. testudinum*, *S. filiforme* (Ugarelli et al., 2018), and *Z. marina* (Ettinger et al., 2017). These bacteria have been described as “dominant and ubiquitous primary surface colonizers in coastal waters” (Dang et al., 2008). Some *Rhodobacterales* groups are known to produce antibacterial compounds that act as a deterrent for other bacteria (Dang et al., 2008; Ettinger et al., 2017), which might explain their persistence and adaptation to different seaweed species and environmental conditions (identified as a major coastal biofilm epiphyte in the Atlantic and Pacific Oceans; Dang and Lovell, 2000).

As for the roots, although statistics show significantly different bacterial communities among the different species, there are only two different groups of differentiator vectors discriminating (pointing toward) the native vs. the invasive species. Most of the discriminating vectors associated with the roots of the native seagrass species were assigned to anaerobic sulfate reducer (SR) bacteria, which are known to contribute to the detoxification of the root area (e.g., *Desulfosarcina*, *Desulfococcus*, and *Desulfarculaceae*), and are also capable of carbon decomposition (Kleindienst et al., 2014). The invasive *Halophila* root differentiation vectors are mostly organic carbon (e.g., *Oceanospirillales*, *Myxococcales*, and *Bacteroidales*) and hydrocarbon (e.g., *Oleibacter*) degraders usually found associated with seagrasses roots and rhizosphere (Crump and Koch, 2008; Ettinger et al., 2017; Gribben et al., 2017; Ugarelli et al., 2017, 2018; Cúcio et al., 2018; Martin et al., 2018) and the order *Chromatiales* was the only SR bacterial vector. The results suggest that, most likely, different bacterial communities (especially the core microbiome associated with native species vs. invasive species) are performing the same functions that have already been shown for the core microbiome of some coral species (Hernandez-Agreda et al., 2018). However, the order *Oceanospirillales* (in our specific case belonging to the *Halomonadaceae* family) is also a halotolerant/halophilic taxon (Franchini et al., 2014) and might play a role in *H. stipulacea* adaptation by conferring some salt tolerance to the host plant (Ruppel et al., 2013). That evidence is strengthened by the fact that the order *Oceanospirillales*, in our core analysis results, appears exclusively and in high abundances, not only in *H. stipulacea* roots but also in leaves.

As discussed above, we found a species-specific pattern among the bacterial communities of the three seagrass species. Additionally, we would also expect to find a more similar bacterial community structure between the native species, which evolved together in the same environment, compared to the invasive species that only recently arrived in that environment (Aires et al., 2015). Yet, our results were rather ambiguous; the core community analysis showed a higher similarity between the invasive *H. stipulacea* and the native *H. wrightii* sharing 27 OTUs (corresponding to 14 genera) against the native species which only shared seven OTUs (corresponding to 5 genera), while our differential analysis shows that native species have fewer differential genera (97) than invasive vs. native species (*H. stipulacea* vs. *T. testudinum* = 312, *H. stipulacea* vs. *H. wrightii* = 138). However, it is important to highlight that the depicted Venn diagram was drawn using the core OTU table and not the total OTU table. The core microbiome, besides its ubiquity, has been described as having essential functions, to a certain type of organism community, ranging from nutrition to protection against diseases (Shade and Handelsman, 2012). As such, the core bacteria have also been described as potential symbionts due to their tightness and persistence across different species (Hernandez-Agreda et al., 2018). That could mean that sharing a higher number of that small group of highly persistent OTUs, *Halophila* and *Halodule*, may reflect more similar needs (e.g., nutritional, adaptive, and physiological) that could be considered essential to the plants, in contrast with the two natives that might share more environmentally responsive community (Hernandez-Agreda et al., 2018). One could also argue that these two species could have exchanged more bacteria during the invasion process but, in our sampling, *Halophila* and *Halodule* were only sampled together in two sites and always together with *Thalassia*, which makes it less likely to have a higher exchange opportunity than *Halophila* and *Thalassia*.

The native and invasive species separated by a large number of differential genera (mostly enriched in the native species, relative to the native) could be explained by the fact that they did not evolve in the same environment, not sharing the same original “bacterial source” which contributed for a higher differentiation. Native species differentiation showed a more conspicuous separation by tissue than by species, which was already discussed above.

Several functional hypotheses of microbial roles can be suggested for the euryhaline capacity of *H. stipulacea*, a species able to invade different environments mainly due to its tolerance to a wide range of salinities (den Hartog, 1970; Oscar et al., 2018; Por, 1971; Winters et al., 2020). Unlike most of the seagrasses to which the salinity limits range from 20 to 40 PSUs (Touchette, 2007), *H. stipulacea* thrives in a wide range of salinities that can go up to 60 PSUs (Oscar et al., 2018). The capacity to survive and grow in those high salinities has been associated with its invasive success and advantage over the seagrasses it has been displacing [see review by Winters et al. (2020)]. The interaction between plants and their associated microbiomes play an important role in plant adaptation to new environments and to tolerate stress conditions (Hardoim et al., 2008; Rosenberg and Zilber-Rosenberg, 2011), raising the hypothesis that part of *H. stipulacea*’s tolerance to salinity could be attributed to its associated microbes (Oscar et al., 2018). Based on the review of Etesami and Beattie (2018), where several halophytic plants were mined for halotolerant PGP bacterial genera, we have selected genera to look for in the three studied seagrass species. The invasive *H. stipulacea* has shown a much higher relative abundance of most of the considered halotolerant bacteria, with PGP abilities, which may be a clue of the importance of these bacterial phylotypes for this species (Ruppel et al., 2013; Etesami and Beattie, 2018). Several studies have investigated halophyte species with the perspective of seeking for potential sources of halotolerant bacteria with PGP potential (Ramadoss et al., 2013; Sharma et al., 2016). These would be further used as an alternative strategy to, for example, enhance crop salt tolerance while boosting crop growth (Dodd and Pérez-Alfocea, 2012). The genera found to be more abundant in the invasive species have growth-promoting properties that range from N2 fixation, indole-3- acetic acid (IAA) production, siderophore production, phosphate solubilization, and 1-Aminocyclopropane-1-Carboxylate (ACC) deaminase activity. Only some will be discussed next. The genus *Halomonas* has been widely recognized for its important growth-promoting traits such as (i) the ability to provide phosphorus to the plant under P-limiting conditions; (ii) the production of IAA-like phytohormones, providing Fe to plants through chelation and uptake (siderophores); and (iii) ACC deaminase production, which lowers ethylene (plant stress hormone) levels and promotes plant growth and development under adverse environmental conditions (Etesami and Beattie, 2018; Gupta and Pandey, 2019). Our analysis shows a much higher relative abundance of *Halomonas* in the invasive species compared to the native ones. This may be an indication that these bacteria could be helping to mitigate the osmotic stress in extreme (or discrepant) salinities and, therefore, contributing to *H. stipulacea* invasiveness. Furthermore, other highly abundant genera associated with *H. stipulacea*, *Salinimonas*, and *Salinicola*, have been recognized as producing quorum sensing (QS) inhibitors (Romero et al., 2012; Tang et al., 2013). QS is one of the pathogenic bacteria processes that allow to reduce host immune response and the establishment of infection (Reading and Sperandio, 2006). Therefore, inhibition of QS could be increased in *Halophila* and so alleviating the disease pressure, which will be an advantage compared to the native species and concomitantly promote its successful spreading. As for the halotolerant PGP genus that occurred exclusively in *H. stipulacea*, *Lysinibacillus*, besides some of the properties mentioned above, was also found to be cadmium and lead tolerant, removing them from the soil and mitigating the heavy metal stress (Pal and Sengupta, 2019). *Rhodococcus* and *Azospirillum* were present in an extremely high proportion in the invasive species compared to the native ones (where their proportion was close to 0); these species were found to greatly increase germination of plants in high salinity conditions (Rueda-Puente, 2017). Despite the strong indications that *H. stipulacea* could be, indeed, taking advantage from the high abundance of salinity-tolerant PGP, only the isolation of these specific phylotypes with posterior inoculation experiments would give us a solid answer about the potential of these bacteria. We stress that our list of halotolerant bacteria only considered those reported in Etesami and Beattie reviews (Table 1). As such, we did not account for other halotolerant bacteria in our dataset, or other bacteria that could have the same or similar functions (salt tolerant with PGP abilities) and were not documented yet.

### Prediction of the Functional Role of the Bacterial Community Members

From the predicted pathways found, most were not relevant for conferring a possible advantage to the invasive species (considered mostly “housekeeping functions”), and some were plant-specific and not likely found in bacteria. For example, *Halophila* roots have shown a higher abundance of biosynthesis of isoflavonoids; these are plant secondary metabolites known to play an important role in adaptation to their environment, both as defensive and as chemical signals in symbiotic nitrogen fixation with rhizobia (García-Calderón et al., 2020) and are not produced by bacteria. That means that there is a low representation of those pathways that could possibly bring advantage to the seagrasses, enhancing its importance as possible assets for the invasion facilitation. The putative functional traits that could possibly bring some advantage to the invasive species were differentially found in *H. stipulacea* roots and were lipoic acid metabolism; glycerine, serine, and threonine metabolism; and hypotaurine and taurine metabolism. Lipoic acid metabolism has been associated with protecting plants against oxidative stress and helping on detoxification (Navari-Izzo et al., 2002). Glycerine, serine, and threonine metabolism pathways have been described as important processes in plants by linking nitrogen and carbon metabolisms and helping to maintain the redox balance and energy levels in stress conditions (Igamberdiev and Kleczkowski, 2018). High levels of hypotaurine have been correlated with high sulfur oxidation and its primary function was assigned to sulfide detoxification in marine invertebrates (Rosenberg et al., 2006); this organic osmolyte may have the same role in plants. Also, it has been shown that taurine promotes the growth of wheat seedling and increased root length (Hao et al., 2004). The leaves of *H. stipulacea* were also found to have some differentially associated putative advantageous functions such as the biosynthesis of staurosporine that have been implied in the protection against some plant fungal and other pathogens (Park et al., 2006).

All these ideas, as well as all our discussion around the putative functions of some particular bacterial phylotypes, are merely hypotheses, based on literature research and a predictive algorithm (Tax4Fun). These hypotheses are raised as suggestions for future testing by meta-omic analyses and manipulation experiments with bacterial isolates, especially to verify if the associated bacterial community actually confers some adaptive advantage to the invasive *H. stipulacea*.

### Conclusion

This study revealed that bacterial communities are different between the three seagrass species in Curaçao, where the invasive species holds a more diverse bacterial community compared to the native ones, with three times more species-specific OTUs. Bacterial community structure follows a species-specific pattern with some influence of the environment (sampling location), and that pattern holds for both leaves and roots. Despite showing significant differences among seagrasses, the root microbiome is less structured, especially for both native species, which shared a higher number of phylotypes, showing a higher influence of the surrounding sediment, which could facilitate microbiome sharing. For those communities that are tightly connected to the seagrasses, likely performing essential functions (“core community”), there is a higher similarity between the invasive *H. stipulacea* and the native *H. wrightii*, which could mean that these two species have a more similar organizational and physiological structuring (similar essential functions) relying on similar core-associated bacteria. The bacterial taxa that are, differentially, more closely associated with the invasive species, along with the highly abundant halotolerant/halophilic bacteria, have been described in the literature to possibly perform beneficial functions, such as growth promotion, epiphyte degradation, salinity tolerance, detoxification, nutrient provision, and QS inhibition, which could help to prevent diseases. Besides, predicted functional profiles have also revealed that the invasive species has a higher number of differentiating putative KEGG pathways, especially at the root level, as compared to the native species, from which only a few could be considered advantageous to the invasive species by bringing possible protection against pathogens, helping in detoxification and increasing stress tolerance. All these are predictions and should not be taken as certain but instead used to formulate a new hypothesis and open paths to a more detailed experimental investigation about seagrass microbiome function and their role in the invasion process. However, it is important to keep in mind that these differences may also be related to physiological and morphological differences among seagrass species. In order to assess this hypothesis, further analysis using meta-omics analyses should be done, linked to experimental manipulations that could provide evidence on the role of these functional differences. In the case of *H. stipulacea*, we suggest a future comparison of the bacterial community from its native range with that from both the Mediterranean and Caribbean habitats where this species has a contrasting invasion success. Nevertheless, this investigation provides novel insights, information, and clues on native vs. invasive seagrass microbiomes that fill in this knowledge gap and guide more in-depth investigations on how associated microbiomes might influence the invasion process.

## Data Availability Statement

The datasets generated for this study can be found in online repositories. The names of the repository/repositories and accession number(s) can be found in the article/Supplementary Material.

## Author Contributions

AE: conceptualization, sampling, and supervision. AE and TA: methodology. TA and TS: laboratory work, *in silico* analysis, and writing – original draft preparation. TA, TS, GM, ES, and AE: writing and review and editing. GM and ES: funding acquisition. All authors contributed to the article and approved the submitted version.

## Conflict of Interest

The authors declare that the research was conducted in the absence of any commercial or financial relationships that could be construed as a potential conflict of interest.

## Publisher’s Note

All claims expressed in this article are solely those of the authors and do not necessarily represent those of their affiliated organizations, or those of the publisher, the editors and the reviewers. Any product that may be evaluated in this article, or claim that may be made by its manufacturer, is not guaranteed or endorsed by the publisher.

## Footnotes

1
www.mrdnalab.com

2
www.arb-silva.de

3
https://www.mg-rast.org/linkin.cgi?project=mgp91806
