# Supplementary figures and images for: Characterization and Comparison of Bacterial Communities of an Invasive and Two Native Caribbean Seagrass Species Sheds Light on the Possible Influence of the Microbiome on Invasive Mechanisms
Source: Front Microbiol. 2021 Aug 3;12:653998. doi: 10.3389/fmicb.2021.653998 (PMC8381869; doi:10.3389/fmicb.2021.653998)

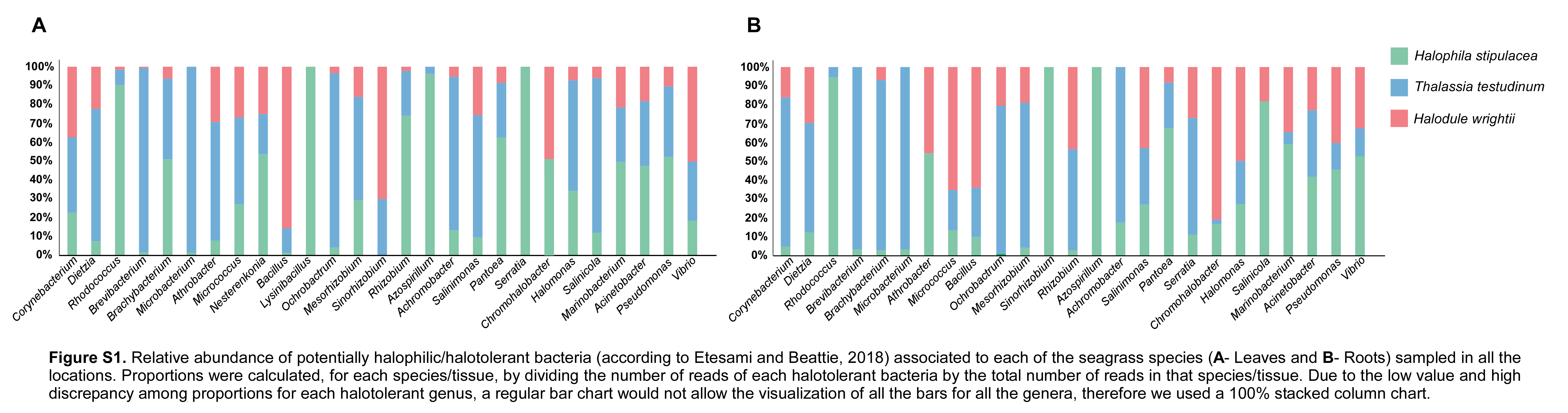

Supplement: Supplementary file 1 [file Image_1.TIF]
